# Supplementary material for: 2-Photon imaging of fluorescent proteins in living swine
Source: Sci Rep. 2023 Aug 29;13:14158. doi: 10.1038/s41598-023-40638-z (PMC10465491; doi:10.1038/s41598-023-40638-z)
Supplement: Supplementary file 1 — Supplementary Information. [file 41598_2023_40638_MOESM1_ESM.pdf]

2-Photon imaging of fluorescent proteins in living swine

Beth A. Costine-Bartell[1], Luis Martinez-Ramirez[1], Kieran Normoyle[2], Tawny Stinson[1], *,#Kevin J. Staley[2], and *,#Kyle P. Lillis[2].
[1]Department of Neurosurgery, Massachusetts General Hospital and Harvard Medical School, Boston, MA, USA
[2]Department of Neurology, Massachusetts General Hospital and Harvard Medical School Boston, MA, USA

*Corresponding Authors: klillis@mgh.harvard.edu, staley.kevin@mgh.harvard.edu
#These authors contributed equally

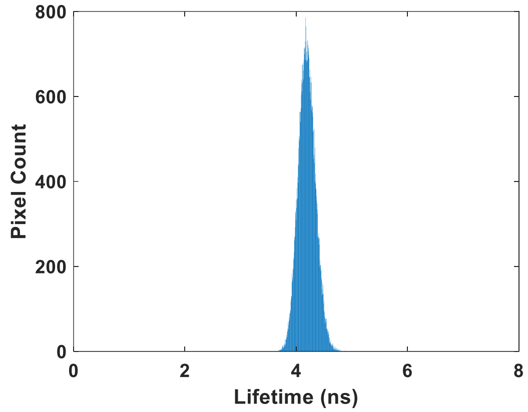

Supplemental Figure 1. Fluorescence lifetime histogram of sodium fluorescein. Our digital FLIM system reported a mean lifetime of 4.197 ns, with a standard deviation of 155ps.

Supplemental Table 1. Conditions for each pig during the problem solving and optimization period. Each pig received a 2 cm burr hole in the skull over the coronal suture about 1 cm from the sagittal suture on each hemisphere: right (R) and left (L). Pigs in gold cells were during the optimization phase where expected distribution of fluorescence was observed; green cells were post-optimization where data was collected.

**Pig #**	**AAV type, titer (gene copies/ mL)**	**AAV injection parameters**	**Dural substitute strategy**	**Age (d) & weight (kg) at AAV inject.**	**Age (d) & weight (kg) at 2P imaging**	**Outcome; any ABP dye injection at imaging.**
81-011	Sclm, 1 x 10^{13}<br><br>GFP $7x10^{12}$	Dura was cut for injection as attempted to inject through the dura and we were afraid of pushing through the dura.<br><br>L: SClm: 1st- 1 uL/min, total = 10 uL, 2nd- 2 uL/min, total 10 uL, 3rd 5 uL/min, total = 10 uL<br>R: GFP: 4th 1 uL/min, total 5.0 uL. Observed blue dye and looked superficial, 5th 5 uL/min, total 5 ul. Observed blue dye coming out of injection site. Needle moved.	otologic repair graft installed	150, 18	171, 29	Significant swelling from window access/ imaging. No fluorescence from AAV observed.<br><br>Brain cutting results: injection dye was probably too deep.
81-053	SClm, 1 x 10^{13}	Dura was cut for injection on both sides.<br><br>L: SClm 10 uL, 5 uL/min * 3 sites<br>R: SClm10 uL, 5 uL/min * 4 sites; a bit deeper; needle came out or bent due to the injector or rig moving.	otologic repair graft	170, 19.5	198	Otologic repair grafts not observed and dura adhered to cortex. No fluorescence from AAV observed.
82-143	SClm, 1 x 10^{13} mCherry, 2.7 x 10^{13}	Dura was cut on sides, injected, and otologic graft installed right after as to not expose the cortex to air for prolonged periods.<br><br>L: 1st SClm, 10 uL/min, total 50ul, 2nd: mCherry 10 ul /min, 50ul total<br><br>R: 3rd mCherry, 10 uL/min, total 50ul, 4th SCLM 10 uL/min, 50 uL total	otologic repair graft installed and dural edges cauterized	162, 19.5	191, 25	Otologic repair graft was able to be removed and scar tissue was removed over part of the injection sites. PDMS was installed in the burr holes prior to imaging: L: straps with straps glued to skull, R: “top hat".<br><br>Putative dim mCherry fluorescence<br><br>Some fluorescence was noted. ABP Dextran was injected (50ul total, 10 ul/min) in both burr holes for imaging. Cortical impact on R over PDMS. Part of PDMS membrane tore. Left: PDMS membrane

						tore at some point during the imaging session.
83-037	SClm , 1 x 10^{13},<br><br>nuclear GFP $1x10^{13}$	Dura was cut on both sides before injection. Closed immediately after injection.<br><br>L: 1st SClm, 10 uL/min, 50 uL total, 2nd nuclear GFP, 10 uL/min, 50 uL total<br><br>R: 1st: nuclear GFP 10 uL/min, 50 uL total, 2nd: SClm 10 uL/min, 50 uL total	The dura was cauterized and glued to skull on both sides. L: Brain was nicked during PDMS membrane placement with suturing approach so we stopped suturing. R: PDMS membrane NOT sutured.	162, 15	192, 23	Left hemisphere PDMS membrane was left in place and worked well. PDMS membrane on right hemisphere would not stay in place and was removed for imaging.<br><br>Some SClm and GFP fluorescence visible on expected channels. Motion artifact apparent, preventing subcellular resolution of SClm.
83-027	SClm, 1 x 10^{13}<br><br>mCherry, 2.7 x 10^{13}	Dura was cut on both hemispheres before the injection. Closed immediately after injection.<br><br>L: 1st: SClm 10 uL/min, total 50ul, 2nd: mCherry, 10 uL/min, total approximately 44 uL.<br><br>R: 1st: mCherry, 10 uL/min, total 50ul, 2nd: SClm, 10 uL/min, total 50 uL	Dura cauterized on both sides and PDMS with "top hat" placed in both burr holes. "Top hat" glued to the skull. Difficult to install the entire PMDS with the "top hat" - caused bleeding of a cortical vessel.			Tonic clonic convulsions day after surgery and animal unable to ambulate. Euthanized early for clinical symptoms and did not image. Large, space filling SDH under cortical window 1 cm thick.<br>In response, we made the "top hat" portion shorter and smaller diameter.<br><br>Brain cutting: depth of injection was ideal.
83-130	mCherry, 2.7 x 10^{13}	Dura was cut on both hemispheres before injection. Closed immediately after injection.<br><br>L: mCherry, 10 uL/min, 50ul total<br><br>R: mCherry, 10 uL/min, total 50ul<br><br>Waiting for new core to make SClm AAV.	Both sides: dura was cauterized, both PDMS ripped when attempting to suture in, learned that small diameter "hat" was too small and removed. One PDMS with tabs.<br>L: PDMS 3.5 cm diameter<br>R: PDMS 3.0 cm diameter	171, 21	198, 29	PDMS membrane in pig left hemisphere adhered to scalp. PDMS on right was cut during access, removed PDMS from both hemispheres, did not replace. After this pig stopped sewing in the PDMS.<br><br>No AAV fluorescence visible<br><br>R: Injected 1X ABP dextran (10 ul/min, total 100 ul) near the previous mCherry injection.<br>L: Injected ABP Dextran (2x) manually (total 100 ul).<br><br>Achieved imaging with ABP dextran.

						After cortical impact in the right hemisphere, the brain appeared indented for some time.
83-142	CAG-tdTomato $1x10^{13}$	Dura was cut on both hemispheres. Closed immediately after injection.<br><br>L: CAG-TDtomato, 10 uL/min, total 45ul<br>R: CAG-TDtomato, 10 uL/min, total 45ul	Both sides: dura cauterized. L: PDMS used just a flat disk with ridges to mark center and glued PDMS to dura. R: PDMS with hat was inserted and glued to skull.			Left hemisphere cut when accessing PDMS window. After this we added a skull retention ring to protect the PDMS window when accessing. Flat PDMS glued to dura worked best. Easier to install than tabs and top hat and remained in place.<br><br>Moderate tdTomato fluorescence observed on expected channel.<br><br>ABP dextran injected. Obtained data for pre- and post-cortical impact.
85-071	N/A	N/A	PDMS inserted at dye imaging	N/A	149, 20	Injected 3X ABP Dextran (100ul total, 500ul/min)<br>Obtained data for pre- and post-cortical impact.
85-072	N/A	N/A	PDMS inserted at dye imaging	N/A	156, 17	PDMS membrane installed. L 3x ABP Dextran 50 ul/min, total 100ul<br>R: ABP Dextran 50ul/min, total 100ul<br>Obtained data for pre- and post-cortical impact.
85-144	N/A	N/A	PDMS inserted at dye imaging	N/A	131, 18	ABP dextran injected. Obtained data for pre- and post-cortical impact.
86-026	SClm, $8.9x10^{13}$	AAV injected through PDMS immediately after PDMS installation.<br><br>L: SClm 10 uL/min, total 50uL<br>R: SClm 10 uL/min, total 50uL	Both sides installed one at a time, PDMS: 3.0-3.5 cm disc with ridges to mark center glued to dura. Installed skull retention ring.	135, 20	156, 26	Worked well, obtained good data.
86-027	SClm, $8.9x10^{13}$	AAV injected through PDMS immediately after PDMS installation.	Both sides installed one at a time, PDMS: 3.0-3.5 cm disc with ridges to	139, 19	163, 24	Worked well, obtained good data. SCLM visualized in **Figure 5**.

		L: SClm 10 uL/min, total 15 uL * 4 sites<br>R: SClm 10 uL/min, total 15 uL * 3 sites	mark center glued to dura. Installed skull retention ring.			
86-060	SClm, 8.9×10^{13}	AAV injected through PDMS immediately after PDMS installation.<br><br>L: SClm 10 uL/min, total 15 uL * 3 sites<br>R: SClm 10 uL/min, total 15 uL * 3 sites	Both sides installed one at a time, PDMS: 3.0-3.5 cm disc with ridges to mark center glued to dura. Installed skull retention ring.	138, 16	170, 28	Worked well, obtained good data.
86-115	SClm, 8.9×10^{13}<br><br>jRGECO 1×10^{13}	AAV injected through PDMS immediately after PDMS installation.<br><br>L: SClm 10 uL/min, total 14 uL * 3, jRECO 10 uL/min, total 10 uL<br>R: SClm 10 uL/min, total 14 uL * 3, jRECO 10 uL/min, total 10 uL	Both sides installed one at a time, PDMS: 3.0-3.5 cm disc with ridges to mark center glued to dura. Installed skull retention ring.	133, 19	175, 30	Worked well, obtained good data.
86-131	SClm, 8.9×10^{13}<br><br>jRGECO 1×10^{13}	AAV injected through PDMS immediately after PDMS installation.<br><br>L: SClm + jRECO mix 10 uL/min, total 30 uL * 3<br>R: SClm + jRECO mix 10 uL/min, total 30 uL * 3	Both sides installed one at a time, PDMS: 3.0-3.5 cm disc with ridges to mark center glued to dura. Installed skull retention ring.	154, 20	175, 24	Worked well, obtained good data.
80-092	N/A	N/A	PDMS inserted at dye imaging	N/A	479, 86	ABP Dextran injected 10 ul/min, total 50 uL. Worked well, obtained good data.
80-094	N/A	N/A	PDMS inserted at dye imaging	N/A	488, 76	ABP Dextran injected 10 ul/min, total 50 uL. Additional 40 uL injected both sides, manually. Worked well, obtained good data. Noted that left burr hole moved caudally from original site 12 months prior.
